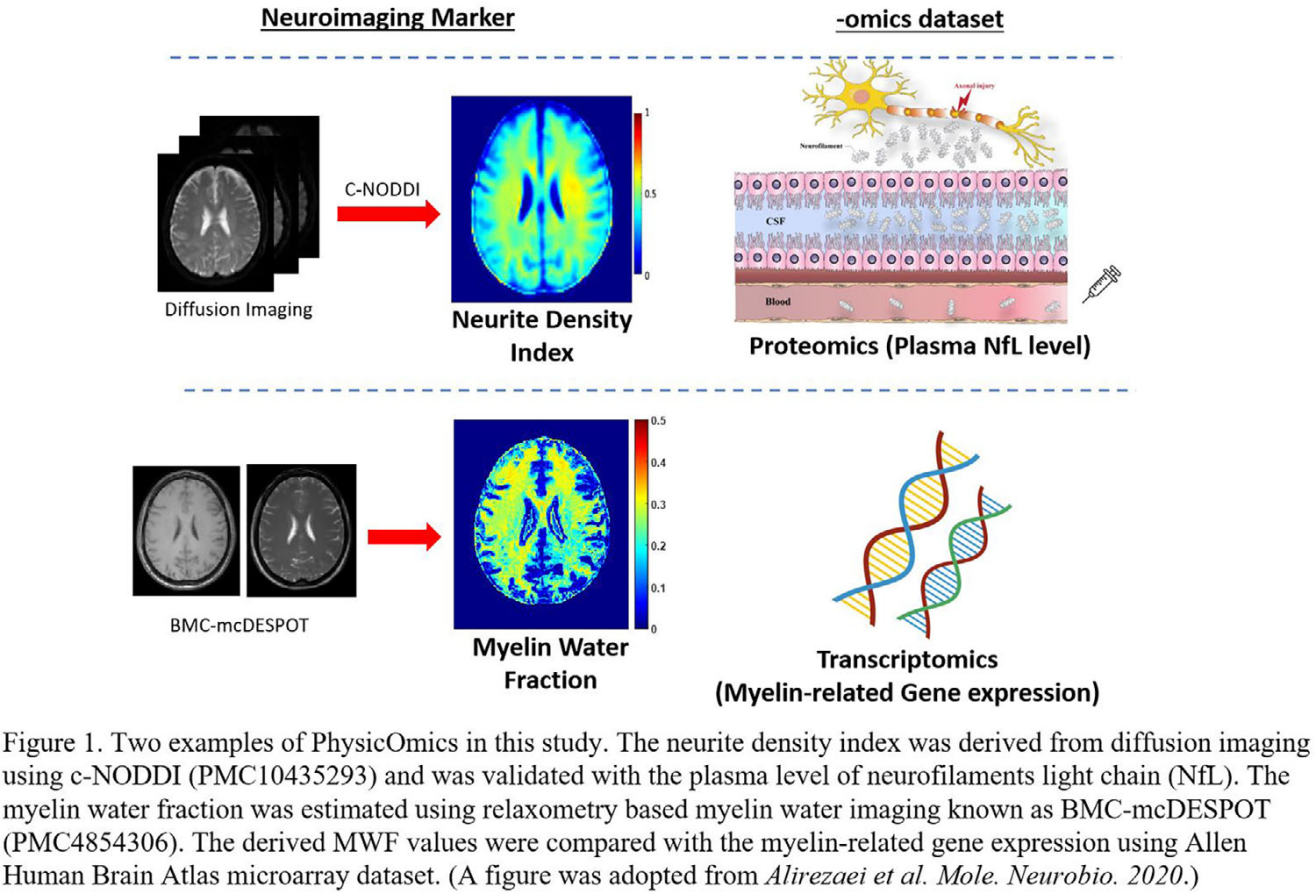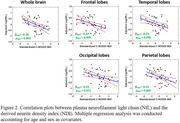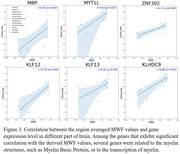# PhysicOmics: Combining MR physics, proteomics, and transcriptomics to study cerebral aging and neurodegeneration

**DOI:** 10.1002/alz.093695

**Published:** 2025-01-09

**Authors:** Jonghyun Bae, Zhaoyuan Gong, Alex Guo, John P Laporte, Mary E Faulkner, Mustapha Bouhrara

**Affiliations:** ^1^ National Institute on Aging, Baltimore, MD USA; ^2^ Laboratory of Clinical Investigation, National Institute on Aging, Intramural Research Program, Baltimore, MD USA

## Abstract

**Background:**

Neuroimaging‐based evidence suggests that changes in cerebral tissue determinants, including axonal density and myelin content, are associated with aging and neurodegenerative diseases. While neuroimaging markers show strong association with physiological changes, direct validation of their specificity remains challenging. Histology provides useful information for validation, however, faces limitations including denaturation of the sample during preparation. However, in the era of omics, validation of neuroimaging markers becomes accessible with dataset from various biological measurements. We provide two examples of validations of our MR physics‐based markers of myelin water fraction (MWF), a surrogate of myelin content, and neurite density index (NDI), a proxy of axonal density, using proteomics and transcriptomics (Fig.1).

**Method:**

Association between neurofilament light chain (NfL) and NDI: 58 participants (age = 21‐83yrs.) underwent our multishell diffusion imaging protocol for NDI determination using C‐NODDI. Blood was collected at the time of MRI to quantify the NfL level, a biomarker of axonal integrity, using Simoa assays. The association between NDI and NfL was evaluated using a multiple linear regression analysis. Association between myelin gene expression and MWF: 132 subjects (age = 22‐94yrs.) underwent our relaxometry protocol for MWF determination using BMC‐mcDESPOT. Further, microarray data were obtained from the Allen Human Brain Atlas, providing gene expression data from 6 healthy donors. Using Pearson correlations, we assessed the association between regional MWF measurements (averaged across participants) and regional gene expressions, with a focus on Myelin Basic Protein (MBP) and transcription factors involved in myelin synthesis.

**Result:**

Figure 2 shows strong negative regional correlations between NfL and NDI derived using C‐NODDI. Further, we found strong positive correlations between MWF and genes involved in myelin production (Fig.3).

**Conclusion:**

Given the strong correlations between NfL or MBP and NDI or MWF, this work provides evidence of the specificity of these MRI biomarkers of axonal density and myelin content, making C‐NODDI and BMC‐mcDESPOT powerful and relevant MR methods for clinical investigations and trials. While further investigations are still required in the context of neurodegeneration, our work demonstrates the potential of combining MR physics and omics to delve deeper into the neurobiological and molecular mechanisms underlying aging and age‐related diseases.